# 

**Published:** 1874-02

**Authors:** 


### CONTENTS:

ORIGINAL COMMUNICATIONS:

ARTICLE I—Staphyloraphy and Uranoplasty. By John E. Owens, M.D., Chicago ........ 65

ART. II.—Annual Report of the Section on Obstetrics and Diseases of Women and Children of the Chicago Society of Physicians and Surgeons. By A. Reeves Jackson, M.D., Chairman of Section ........ 74

ART. III.—Transactions of the Chicago Society of Physicians and Surgeons. Meeting of Dec. 8, 1873. Reported by Plym. S. Hayes, M.D. ........ 94

SELECTIONS:

Cases of Peripheral Paralysis, their Causes and Nature ........ 96

Bloodless Surgery of the Extremities .. 105

Case of Hæmorrhage from the Internal Carotid Artery treated successfully by the Ligature ........ 108

Physiological Influence of Alcohol ..... 113

EDITORS' BOOK TABLE ........ 116

EDITORIAL ........ 125

LOOT ........ 127

